# Retrospective study on antibiotic resistance profiles of uropathogenic *Escherichia coli* with emphasis on β-lactamase production in a tertiary hospital in Saudi Arabia

**DOI:** 10.3389/fmicb.2026.1867292

**Published:** 2026-06-17

**Authors:** Yasser Alraey, Irfan Ahmad, Mushtaq Ahmad Mir, Mohd Saleem, Saleh Abdullah Amer Alshehri, Nawal Mohammad Elkdshani, Ali Alasmari, Menah Halaway, Abdulaziz Saeed Mohammed Alqahtani, Bandar Abdullah Alyazeedi, Awdhesh Kumar Mishra, Abdulah OS Bawazeer, Mohd Shahid Khan

**Affiliations:** 1 Department of Clinical Laboratory Sciences, College of Applied Medical Sciences, King Khalid University, Abha, Saudi Arabia; 2 Department of Pathology, College of Medicine, University of Hail, Hail, Saudi Arabia; 3 Asir Central Hospital, Saudi Ministry of Health, Abha, Saudi Arabia; 4 Central Blood Bank, Aseer Region, Abha, Saudi Arabia; 5 Department of Biotechnology, Yeungnam University, Gyeongsan, Republic of Korea; 6 King Faisal Medical City for the South Region, Abha, Saudi Arabia; 7 Department of Microbiology, Hind Institute of Medical Sciences, Sitapur, Uttar Pradesh, India

**Keywords:** antimicrobial resistance, beta-lactamases, epidemiological trends, MDR, XDR

## Abstract

**Objective:**

Urinary tract infections (UTIs) remain a major public health concern with increasing antimicrobial resistance worldwide. This study aimed to evaluate the demographic distribution, antimicrobial resistance patterns, and trends of multidrug-resistant *Escherichia coli* isolated from UTI cases over 5 years.

**Methods:**

A retrospective analysis was conducted on UTI isolates collected between 2019 and 2023. Demographic data, clinical setting (inpatient vs. outpatient), and antimicrobial susceptibility profiles were analyzed. Phenotypic and molecular detection of major resistance mechanisms was performed for selected isolates.

**Results:**

A total of UTI cases showed a higher prevalence in females (57.5%), with most infections occurring in the elderly age group (61–80 years). A notable shift in healthcare settings was observed, with inpatient cases decreasing over time and outpatient cases increasing, suggesting a rise in community-associated infections. An increasing trend in antimicrobial resistance was observed among *E. coli* isolates, particularly against fluoroquinolones, cephalosporins, and carbapenems, while nitrofurantoin retained comparatively lower resistance rates. A rising proportion of multidrug-resistant (MDR) and extensively drug-resistant (XDR) strains was detected over the study period, with ESBL-producing isolates being the most common resistant phenotype.

**Conclusion:**

The study demonstrates a concerning increase in antimicrobial resistance among UTI pathogens, along with a growing burden of MDR/XDR *E. coli*. These findings highlight the need for continuous surveillance, strengthened antimicrobial stewardship, and effective infection control strategies to limit further spread of resistant uropathogens.

## Introduction

Urinary tract infections (UTIs) represent a significant bacterial infection pathologically found in many clinical environments. Leading the ranking among acquired diseases of the community, they represent the most prevalent type of infectious diseases (Foxman et al., 2000; Foxman, 2010). Epidemiological reports from 2022 identified 400 million UTI cases worldwide and established a death count of 230,000 yearly (Yang et al., 2022), while 35% of nosocomial infections belong to this category, making UTIs the most prevalent hospital-acquired infections. In hospital settings, UTI leads to bacteraemia in the second most common manner (Alanazi et al., 2015). UTIs were frequently reported in Saudi Arabian population statistics since they made up 10% of all reported infectious diseases. Evaluating clinical management with speed remains essential because UTIs are the second leading reason for emergency department patient admissions, causing potentially severe consequences (Alanazi et al., 2015; Alrashid et al., 2022; Flores-Mireles et al., 2015).

Female populations are particularly affected by UTIs, which result in episodes for approximately 60% of all female patients throughout their lives. The anatomical risk factors of women and additional host-specific factors lead to more frequent UTI recurrences than occur in men. The male genital tract creates an ideal physiological condition that promotes the growth of UTIs and subsequent related complications. UTIs most commonly develop because of *Escherichia coli, Klebsiella pneumoniae, Staphylococcus saprophyticus, Enterococcus faecalis*, and *Proteus mirabilis* infection (Sobel and Kaye, 2015). Uropathogenic *E. coli* (UPEC) stands as the dominant infection agent for UTI since it causes between 60 and 90% of all cases. Most UTIs develop in the community rather than the healthcare system, where less than half of all cases originate (Foxman, 2010; Gharavi et al., 2021). UPEC infection likelihood rises because of multiple factors that include heightened sexual relations, together with certain birth control methods, abnormalities of the urinary tract and conditions that suppress immune function (Muriuki et al., 2022; Terlizzi et al., 2017).

Antimicrobial resistance serves as an important danger to public health while resulting in roughly 700,000 yearly deaths among the world population. Predictions suggest that no action to control this figure will result in more than 10 million cases by the year 2050 (O’Neill, 2016). The World Health Organization (WHO) has made developing new antimicrobial agents that target multidrug-resistant (MDR) pathogens with a special focus on extended-spectrum β-lactamase (ESBL)-producing Enterobacteriaceae (ESBL-E) their top priority because of this difficult situation (Tacconelli et al., 2018). Emergency action becomes necessary to control the AMR crisis since ESBL-E prevalence, together with community-acquired infections, has increased by 50% during the last decade (Jernigan et al., 2020).

The problem with rising antimicrobial resistance (AMR) among UPEC strains demands serious attention because the pathogens show increasing resistance to front-line antibiotics such as fluoroquinolones, cephalosporins, and aminoglycosides. Mutant bacterial resistance factors that contain antibiotic resistance determinants are responsible for this resistance phenomenon (Bartoletti et al., 2016; Kot, 2019; Sanchez et al., 2016; van Driel et al., 2019). UPEC utilizes multiple virulence components to colonize urinary tract epithelial cells through fimbria attachments and implements iron acquisition methods to endure urinary tract iron scarcity, together with flagella-powered mobility and toxin-producing capacities for pathogenicity. The virulence determinants present as genetic elements that exist on either plasmids or chromosomal accessory elements, which enable bacteria to obtain pathogenic traits from other bacterial populations through horizontal gene transfer (Farshad et al., 2012; Johnson et al., 2005).

MDR UPEC strains, together with ESBL-producing isolates, present serious challenges for effective clinical treatment (Adamus-Białek et al., 2018; Amabile-Cuevas, 2021). The treatment of ESBL-UTI with either improperly matched or belated antibiotic choice results in worse clinical results that elevate sepsis risk, damage the kidneys, and keep patients in hospitals longer than typical non-ESBL patients (Abernethy et al., 2017; Ling et al., 2021; Naziri et al., 2020; Thenmozhi et al., 2014). The worldwide incidence of *E. coli* strains producing ESBLs keeps increasing, although geographical differences in resistance patterns exist (Khanfar et al., 2009; Shakya et al., 2017). Standardized criteria must exist for bacterial isolate selection in order to accurately measure ESBL infection rates (Jia et al., 2021; Mohammed et al., 2016). The ESBL enzymes can break down multiple categories of β-lactam antibiotics, which contain penicillins and cephalosporins as well as monobactams, including aztreonam (Jia et al., 2021; Khanfar et al., 2009; Mohammed et al., 2016; Shakya et al., 2017). The combination of MDR UPEC strains causes treatment difficulties due to their resistance against cotrimoxazole, quinolones, and aminoglycosides (Pandit et al., 2020).

Detection of beta-lactamase-producing uropathogens at an early stage, along with perfect identification, remains vital because it helps optimize antimicrobial choices and develop efficient infection control approaches. The challenge of choosing suitable first-choice antibiotics proves the need to maintain ongoing testing of resistance trends in bacterial populations. Various studies demonstrate how extensive antimicrobial susceptibility data analysis becomes necessary for developing proper empirical therapy while also fighting against AMR (Emody et al., 2003; Jia et al., 2021; Justice and Hunstad, 2012). A medical examination investigates the antibiotic resistance profiles of *E. coli* isolates recovered from urinary tract infection patients in a Saudi Arabian tertiary care hospital setting. The study results will yield important data about resistance behaviors to support evidence-informed treatment procedures that boost treatment effectiveness and infection prevention protocols.

## Materials and methods

### Study design

Retrospective observational research evaluated 3,623 *E. coli* isolates obtained from clinical specimens collected at a tertiary referral and teaching hospital based in Abha, which serves as the administrative capital of the southern Saudi Arabian Aseer province. Primary healthcare services within the facility receive direct patient referrals and accept other regional healthcare referrals throughout the area. Researchers examined an expertly designed database consisting of entire patient records with confirmed clinical significance obtained from January 2019 to December 2023.

### Data acquisition

The study used a systemized approach for extracting data to maintain procedural consistency while decreasing biased selection. The data collection process included records that contained patient demographics alongside laboratory results and microbiological test outcomes, as well as *E. coli* isolate susceptibilities against antibiotics.

### Ethical compliance

The Institutional Review Board approved this study ethically under permission number REC-E7-2-2025. Granting informed consent, permission came from the hospital administration to access patient medical files under restricted terms. The specific group of researchers received restricted data access for research purposes only after the implementation of established confidentiality policies. Ethical practices, along with non-maleficence principles, successfully safeguard patient welfare, together with patient confidentiality protection.

#### Specimen collection and processing

In this study, clinical specimens were collected from hospitalized patients with laboratory-confirmed *Escherichia coli* infections. To ensure that each case represented a distinct infection episode, only the first *E. coli* urine isolate per patient was included. Inclusion criteria required complete demographic, clinical, and microbiological data, such as patient age, gender, hospital ward, and antimicrobial susceptibility profiles.

To maintain data accuracy and avoid redundancy, duplicate isolates from the same patient were excluded following standard de-duplication protocols for infection surveillance (Clinical and Laboratory Standards Institute [CLSI], 2023). Urine cultures showing polymicrobial growth or signs of contamination, such as mixed flora without a predominant organism or colony counts below thresholds indicative of infection, were excluded. Patients with missing or incomplete clinical or laboratory data, as well as those with *E. coli* isolates from non-urine sources (e.g., blood or wound swabs), were also excluded from the analysis.

Urine was selected as the primary specimen type, as *E. coli* is the predominant uropathogen in both community-acquired and healthcare-associated urinary tract infections (Flores-Mireles et al., 2015). Samples were collected from various hospital departments to ensure a broad epidemiological representation of UTI cases.

#### Specimen collection techniques

##### Midstream clean-catch urine (MSU)

For non-catheterized patients, midstream urine was collected using the clean-catch method, which is widely recommended for minimizing contamination with urethral flora (Clinical and Laboratory Standards Institute [CLSI], 2023; Leber, 2023). Patients were instructed in proper perineal cleaning and instructed to void the initial stream, collecting the midstream portion into sterile, wide-mouth containers.

##### Catheterized urine collection

For patients with indwelling catheters, urine samples were aseptically aspirated from the catheter port using sterile technique, as per the Centres for Disease Control and Prevention (CDC) guidelines (CDC/NHSN, 2023). Samples were never collected from the drainage bag to avoid colonization-related contamination.

##### Suprapubic aspiration (if applicable)

In select pediatric or complex adult cases where contamination risk was high and reliable midstream clean-catch or catheterized urine samples could not be obtained (e.g., non-toilet-trained infants, uncooperative children, or adults with significant clinical conditions precluding proper sample collection), suprapubic bladder aspiration was performed under sterile conditions by trained clinicians. This technique, though invasive, is considered the gold standard for obtaining uncontaminated urine specimens and was used to ensure diagnostic accuracy in such cases (Leber, 2023).

##### Sample transport and processing

Specimens were transported to the microbiology laboratory within 2 h of collection. When delays were unavoidable, samples were stored at 4°C and processed within 24 h to preserve microbial viability and minimize overgrowth (Leber, 2023).

### Bacterial isolation and identification

Microbiological procedures according to standard methods processed the clinical specimens. Medical professionals cultured urine samples through CLED agar before subjecting them to 37°C temperature incubation over 24–48 h. The Vitek-2 automated system from bioMérieux (Marcy-l’Étoile, France) determined *E. coli* species with exact species-level confirmation.

### Antibiotic susceptibility profiling

Antimicrobial susceptibility testing (AST) was performed using the Vitek 2 automated system (bioMérieux, Marcy-l’Étoile, France). Dedicated AST cards, including N291, N292, and N204, were employed for profiling. Minimum inhibitory concentrations (MICs) were generated within 24–48 h, and results were interpreted according to the latest Clinical Laboratory Standards Institute [CLSI] 2020 breakpoints to ensure accuracy and consistency (Clinical and Laboratory Standards Institute [CLSI], 2020; Ferraro, 2001). MDR was defined as non-susceptibility to at least one agent in three or more antimicrobial categories, whereas XDR isolates were defined as non-susceptibility to at least one agent in all but two or fewer antimicrobial categories (Magiorakos et al., 2012).

#### Phenotypic detection of extended-spectrum beta-lactamase (ESBL) using double disk synergy test

All screening-positive isolates for ESBL production, as defined by the CLSI based on resistance to third-generation cephalosporins (ceftazidime, ceftriaxone, cefotaxime, and cefpodoxime), were subjected to phenotypic confirmation using the double-disk synergy test. Briefly, bacterial suspensions were adjusted to 0.5 McFarland turbidity and uniformly inoculated onto Mueller–Hinton agar (MHA) plates using a sterile swab. After allowing the plates to dry for 15 min, ceftazidime (30 μg) and ceftazidime/clavulanic acid (30/10 μg) disks were placed on the agar surface. The plates were incubated at 37°C for 18–24 h. Isolates were considered ESBL producers when the inhibition zone around the ceftazidime/clavulanic acid disk was at least 5 mm greater than that around the ceftazidime disk alone. *Klebsiella pneumoniae* ATCC 700603 and *Escherichia coli* ATCC 25922 were used as quality control strains (Khan et al., 2024a; Khan et al., 2024b).

#### Metallo-β-lactamase (MBL) identification using modified carbapenem inactivation method (mCIM) and EDTA-modified carbapenem inactivation method (eCIM)

The exam of carbapenemase production needed 0.1 mL bacterial culture from blood agar plates combined with 2 mL trypticase soy broth (TSB) then a 10 μg meropenem disk at 37°C for a 4-h incubation. An MHA plate received a bacterial spread of *E. coli* ATCC 25922 based on its 0.5 McFarland standard suspension and contained a meropenem disk obtained from the TSB medium. The isolate displayed carbapenemase activity by producing an inhibition area with a diameter of < 15 mm around the meropenem disk, while negative results had zones measuring at least 19 mm. The quality controls were conducted with *K. pneumoniae* ATCC BAA-1705 as the positive control and BAA-1706 as the negative control. The combination of EDTA in eCIM tests enabled experts to identify both metallo-β-lactamases and serine carbapenemases by producing distinctive results through positive MBL detection within ≥ 5 mm inhibition zone expansion and negative results yielding ≤ 4 mm differences that identified serine carbapenemase activities (Ahmad et al., 2024; Ahmad et al., 2025; Clinical and Laboratory Standards Institute [CLSI], 2020).

#### Detection of AmpC β-lactamase using the cefoxitin-cloxacillin double disk synergy test (CC-DDST)

Cloxacillin acts as an inhibitor of AmpC β-lactamase expression in this method. The standardized bacterial liquid was spread uniformly onto an MHA plate before leaving it to dry for 15 min. The agar test surface received both Cefoxitin disks (30 μg) and Cefoxitin/Cloxacillin disks with 30 and 230 μg of antibiotic. The bacterial isolates became AmpC producers when an overnight incubation at 37°C showed the inhibitory zone surrounding the cefoxitin/cloxacillin disk to exceed the cefoxitin disk zone by at least 4 mm. The testing method provided healthcare laboratories with a trustworthy approach to detect AmpC enzyme activity within clinical bacterial isolates according to Alyamani et al. (2017) and Khan et al. (2024b).

#### Molecular characterization of resistance genes in beta-lactamase producing *E. coli*

Laboratory analysis of beta-lactamase-producing *E. coli*, together with other multidrug-resistant organisms, used polymerase chain reaction (PCR) to trace their resistance determinants. The standard method-based design of oligonucleotide primers enabled the specific amplification of genes related to resistance (Adeyemo et al., 2021; Pasanen et al., 2014).

The QIAamp DNA Mini Kits (Qiagen United States) allowed strict execution of DNA genomic extraction procedures according to the manufacturer’s specifications. Researchers used the primers listed in Table 1 for performing ESBL gene amplification as described in Mashaly et al. (2021).

**TABLE 1:** Target resistance genes: oligonucleotide primers.

Gene target	Name	Primer sequence (5’–3’)	Amplicon size (bp)	References
TEM	TEM-F	ATGAGTATTCAACATTTCCGTG	404 bp	(Adeyemo et al., 2021)
	TEM-R	TTACCAATGCTTAATCAGTGAG		
VIM	VIM-F	ATTCCGGTCGGMGAGGTCCG	530 bp	(Adeyemo et al., 2021)
	VIM-R	GAGCAAGTCTAGACCGCCCG		
NDM	NDM-F	GACAACGCATTGGCATAAG	447 bp	(Adeyemo et al., 2021)
	NDM-R	AAAGGAAAACTTGATGGAATTG		
CTX-M	CTX-M-F	TTGCGATGTGCAGTACCAGTAA	754 bp	(Adeyemo et al., 2021)
	CTX-M-R	CGAATATCGTTGGTGGTGCCATA		
OXA48	blaOXA48-F	GCTTGATCGCCCTCGATT	238 bp	(Gurung et al., 2020)
	blaOXA48-R	GATTTGCTCCGTGGCCGAAA		
KPC	KPC-F	ATGTCACTGTATCGCCGTCT	893 bp	(Adeyemo et al., 2021)
	KPC-R	TTTTCAGAGCCTTACTGCCC		
SHV	SHV-F	ATTTGTCGCTTCTTTACTCGC	294 bp	(Adeyemo et al., 2021)
	SHV-R	TTTATGGCGTTACCTTTGACC		

Each PCR amplicon required agarose gel electrophoresis using a 5 μL volume and 1.5% agarose solution (Biomatik, Kitchener, Ontario, Canada) in 1X Tris-acetate-EDTA (TAE) buffer. The electrophoresis process required 45 min to measure precise sizes of the detected fragments. The reference points for accurate size estimations in electrophoresis were obtained through molecular weight markers ranging from 100 to 1,200 base pairs. Analysis through this method enables scientists to identify precise AMR genes which reveal genetic basis for resistance across multiple MDR bacterial strains.

#### Data analysis

The data processing involved Statistical Package for the Social Sciences (SPSS), version 26 (IBM Corp., Armonk, NY, United States as our data evaluation tool for descriptive analysis, which presented study variables using frequency distribution alongside percentages. Line graphs tracked the case and resistance rate patterns from 2019 to 2023, while bar charts evaluated distribution patterns.

## Results

### Gender distribution

**FIGURE 1:**
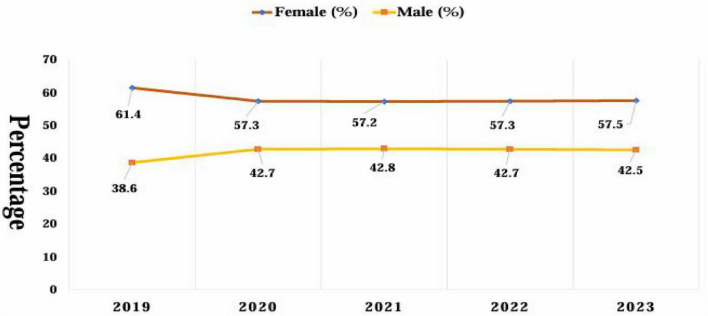
Distribution of *E. coli* among males and females during the study period of 2019–2023.

Over the 5-year study period, females consistently accounted for a higher proportion of UTI cases than males. In 2019, females represented 427/696 cases (61.4%) compared to 269/696 males (38.6%). Similarly, females accounted for 57.3% (296/517) of cases in 2020, 57.2% (445/778) in 2021, 57.3% (431/752) in 2022, and 57.5% (506/880) in 2023. Overall, UTIs were more frequently observed among females throughout the study period (Figure 1), which is consistent with the known higher susceptibility of women to UTIs due to anatomical and physiological factors that facilitate ascending uropathogenic infections.

### Distribution of *E. coli* among patient categories

**FIGURE 2:**
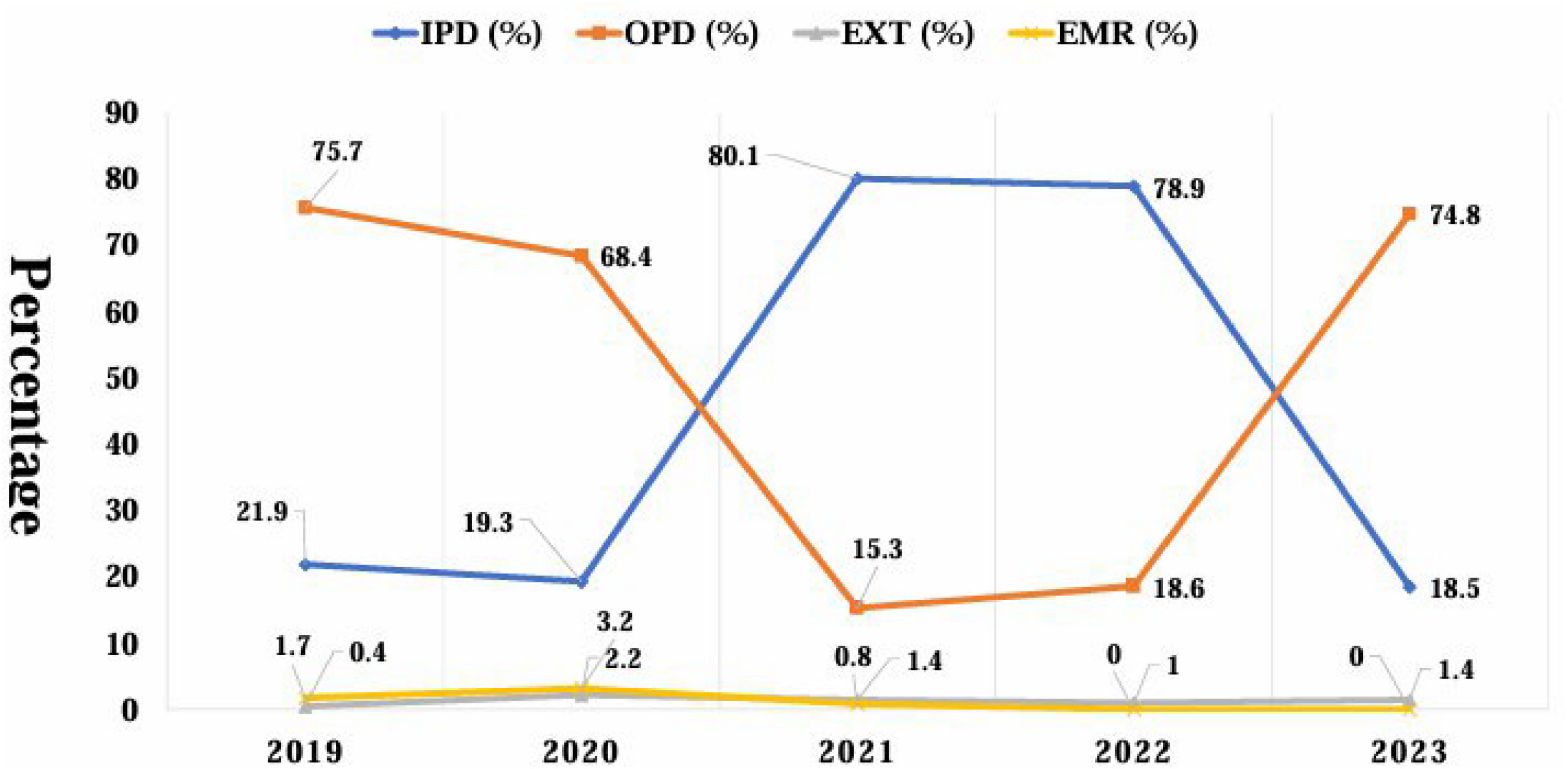
Distribution of *E. coli* among patient categories during the study period of 2019–2023.

The number of admitted patients to inpatient department (IPD) started at 153 (21.9%) in 2019 and decreased to 107 (19.3%) in 2020 before reaching a peak of 639 (80.1%) in 2021 and maintaining 602 (78.9%) in 2022 before declining to 172 (18.5%) in 2023. The number of patients who visited the outpatient department (OPD) grew from 528 (75.7%) in 2019 to 380 (68.4%) in 2020, until reaching 122 (15.3%) in 2021. In 2022, the number of cases experienced a minimal boost (142, 18.6%); afterward, EXT cases surged dramatically to reach 695 (74.8%) during 2023. The frequency of outside hospital patients showed minimal movement during the study period, as External (EXT) numbers oscillated between 3 in 2019 and a single peak of 13 in 2023. Emergency (EMR) cases showed a notable decrease over time, from 12 in 2019 to 18 in 2020, then declining steadily to 6 in 2021, 0 in 2022, and 0 in 2023. These trends suggest a dynamic shift in patient distribution across different healthcare services over the years, possibly influenced by various epidemiological and healthcare system factors (Figure 2).

### Distribution of *E. coli* among age groups

**FIGURE 3:**
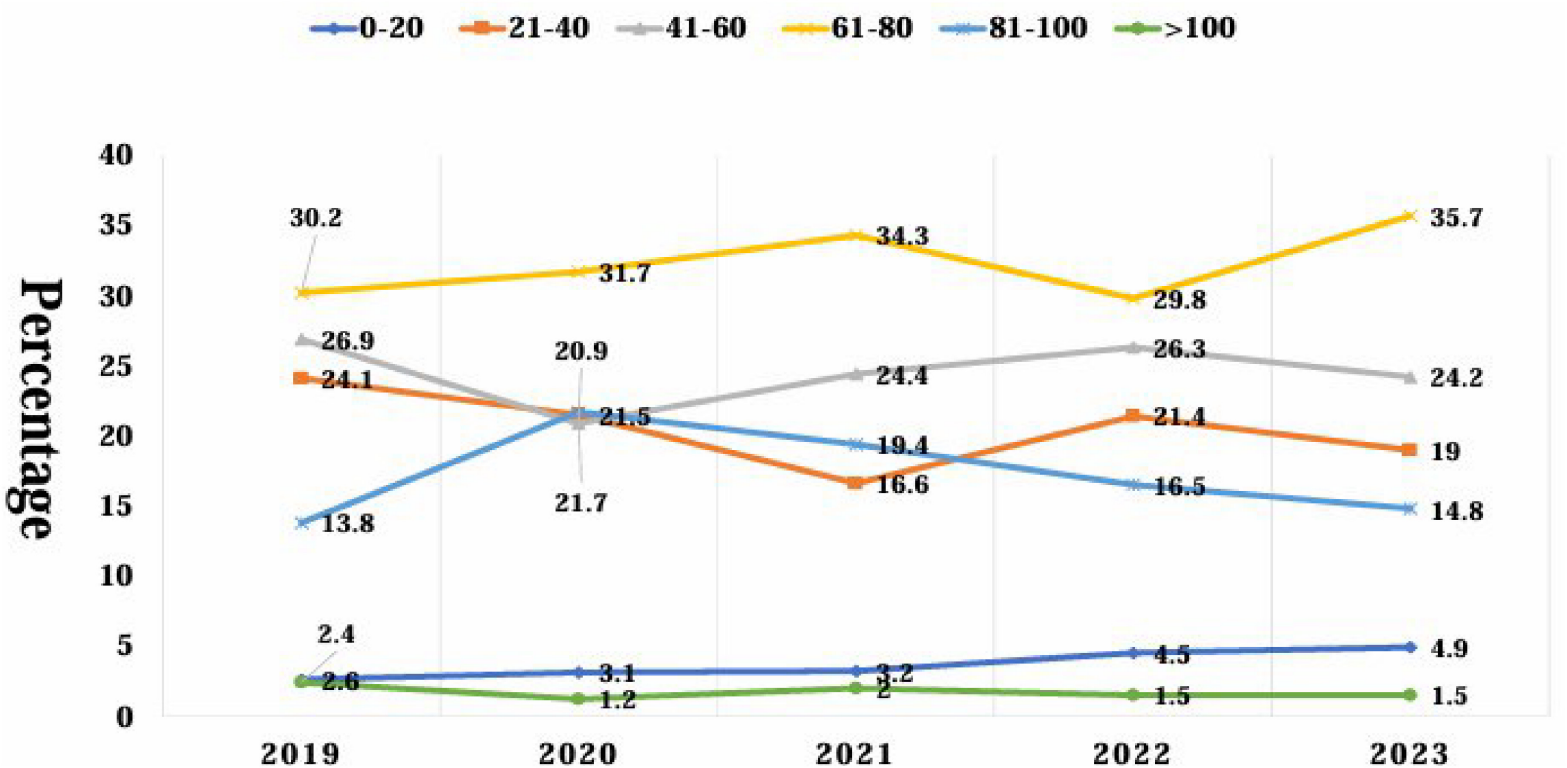
Distribution of *E. coli* among different age groups during the study period of 2019–2023.

The distribution of cases across different age groups from 2019 to 2023 exhibits distinct trends. The 61–80 years demographic recorded 210 (30.2%) cases as the most frequent annual cases, followed by the 41–60 years group with 187 (26.9%) cases, while the > 100 years category presented with the lowest yearly cases total of 17 (2.4%). A reduction in 2020 led to decreases in 41–60 years group members from 108 (20.9%) to 31.7%, and from 164 (31.7%) to 21.7% in the 61–80 years group, resulting in the oldest 81–100 years group becoming the most affected category. The pandemic year of 2021 showed growing numbers of cases among patients aged 61–80 years, reaching 267 (34.3%), as well as the 41–60 years group, who numbered 190 (24.4%). The number of patients in the 61–80 years age group maintained its position as the most affected at 224 (29.8%), and the 41–60 years group followed closely with 198 (26.3%) patients in 2022. Statistics from 2023 showed dramatic growth in the number of cases within the 61–80 years age group to 314 (35.7% of total cases), and the 41–60 years age group reached 213 (24.2%). The percentage of infected patients between the ages of 0–20 years experienced a modest increase from 18 cases in 2019 to 43 cases in 2023 (Figure 3) while maintaining consistently low case numbers throughout all years.

### Antimicrobial resistance pattern of *E. coli* over 5 years

From 2019 through 2023, the development of antibiotic resistance demonstrated significant changes. The percentage of bacteria resistant to imipenem and meropenem elevated progressively from 1.2% in 2019 to 3.1% and from 0.9 to 4.3%, respectively, between 2019 and 2023. From 2019 to 2023, the percentage of Ertapenem-resistant bacteria increased steadily from 2 to 9.4%, thus indicating a reduced effectiveness of carbapenem antibiotics.

The resistance rate for amikacin experienced minimal changes during the period, as it started at 1.3% and elevated to 3.1% in 2023, while gentamicin’s resistance patterns were unstable but kept a steady rate of 16–23%. The annual trend of tobramycin resistance started at 29.7% in 2021 and then descended to 21.9% in 2023. The situation with Cephalosporin resistance demonstrated serious concerns due to increased cefotaxime resistance from 13.5% in 2019 to 32.9% in 2022, followed by a minor decrease to 21.7% in 2023. The resistant pattern of Ceftriaxone showed variations through time, reaching its highest point at 56.4% in 2022 and then descending to 38.7% in 2023. The percentage of organisms resistant to ceftazidime dropped from 37.9% in 2019 to 25.2% in 2023, but cefuroxime resistance substantially increased during this period from 26.6 to 35.3%.

The resistance levels for both ciprofloxacin and levofloxacin stayed consistently high from 2019 to 2023, with ciprofloxacin showing 58–61% resistance and levofloxacin recording 53–58% resistance rates. The drastic uptick in resistance to norfloxacin creates a serious cause for concern, as the rate climbed from 58.4% in 2019 to 97.1% in 2023. The resistance levels of piperacillin-tazobactam among β-lactam and β-lactamase inhibitor combinations increased methodically from 3.5% in 2019 to 7.5% in 2023. The resistance rates of amoxicillin-clavulanate increased from 12.6% in 2019 to 17.7% in 2023. The rate of minocycline resistance reached its highest level at 17.4% in 2020 before declining to 11.1% in 2023. The resistance levels of Tigecycline remained below 2.5% throughout all the examined years. The resistance levels of Trimethoprim-sulfamethoxazole tested between 44 and 47% throughout the 5 years. The resistance rate for nitrofurantoin decreased significantly from 6.8% in 2019 to 1.8% in 2023 (Table 2).

**TABLE 2:** Antibiotic response pattern of *E. coli* during the study period of 2019–2023.

Antibiotics	2019 (S/R/I)	2020 (S/R/I)	2021 (S/R/I)	2022 (S/R/I)	2023 (S/R/I)
AK	93.1/1.3/5.6	93.1/2.5/4.4	90.1/4.4/5.5	88.9/3.3/7.8	89.3/3.1/7.6
AMC	66.2/12.6/21.2	72.2/10.5/17.2	69.5/12.6/17.9	69.2/13.9/16.9	65.9/17.7/16.3
A/S	36.4/39.7/23.9	41/37/22	43.4/32.2/24.4	47.6/33.9/18.5	41.6/40.3/18.2
AMP	28.5/71.1/0.4	38.3/60.6/1.1	34.4/64.6/1	26.1/54.7/19.2	28.9/68.6/2.5
AT	72.1/26.8/1.1	72.3/26/1.7	71.7/22.6/5.7	67.4/22.6/10	72.2/17.8/10
CZ	97.7/1.8/0.6	99.3/0.7/0	96.4/3.6/0	93.8/6.2/0	89.8/10.2/0
CPM	60.7/39.3/0	74.8/23.2/1.9	74.5/18.3/7.2	76.1/17.2/6.7	72.2/21.9/5.9
CTR	39/60/1	46.8/53.2/0	46.3/49.3/4.5	43.6/56.4/0	57.3/38.7/4
CTX	85.2/13.5/1.3	81.5/15.2/3.3	75.7/22.1/2.2	64.4/32.9/2.7	78.3/21.7/0
CX	83.4/11.5/5.1	84.5/12.2/3.3	81/12/6.9	81.8/14.5/3.7	73.9/23.9/2.2
CAZ	60/37.9/2.1	70.6/28.1/1.3	71.1/26.9/2	71/27.3/1.6	72.7/25.2/2.2
CXM	69.7/26.6/3.7	72.5/22.7/4.9	72.3/25.3/2.4	71.1/25.2/3.8	60.6/35.3/4
CIP	38.1/60.7/1.2	39.7/58.6/1.7	37.2/61.4/1.4	36.4/61.1/2.6	.37.1/59.5/3.4
CL	96.6/3.4/0	54.5/45.5/0	69.2/26.9/3.8	85.7/0/14.3	12.2/17.1/70.7
ETP	96.6/2/1.4	93.3/4.9/1.8	92.2/5.8/2	90.3/7.7/2	89.4/9.4/1.2
GEN	75.7/23/1.3	81.8/16.6/1.5	77.1/21.2/1.7	81.1/16.2/2.7	82.3/16.1/1.6
FO	95.5/4.5/0	97.2/2.8/0	100/0/0	0/0/0	77.2/22.8/0
IMP	97.6/1.2/1.2	97.1/1.2/1.6	96.4/2.5/1.2	96.8/1.5/1.8	95.4/3.1/1.5
LE	38.7/58.4/3	40.7/54.7/4.6	37.4/57.5/5.1	37.4/54.5/8.1	38.2/53.7/8.1
MRP	98.4/0.9/0.7	97.5/2.3/0.2	94.6/4.4/1	93.3/4.2/2.5	94.7/4.3/1
MIN	83.8/8.1/8.1	65.2/17.4/17.4	76/13.5/10.4	82.7/6.9/10.4	78.7/11.1/10.1
MOX	31.7/65.3/3	32.3/62.5/5.1	32.4/62/5.5	34.7/56.4/8.9	33.8/56.9/9.3
NIT	88/6.8/5.2	95.7/3.1/1.2	87.8/6.4/5.8	89.1/6.4/4.5	90.8/1.8/7.5
NOR	41/58.4/0.6	41.5/58.5/0	31.2/67.3/1.5	3.5/96.5/0	1.9/97.1/1
PIT	93.2/3.5/3.3	93.4/2.9/3.7	92.6/3.9/3.5	91.9/5.2/3	88.1/7.5/4.4
TGC	99.4/0.4/0.1	97.2/1.2/1.6	96.7/2.3/1	97.4/1.9/0.7	97.3/1.6/1.1
TOB	68.1/27.2/4.7	73.6/23.4/3	65.9/29.7/4.4	74.5/20.7/4.9	74.4/21.9/3.7
COT	52.4/47.6/0	55.7/44.3/0	52.9/47.1/0	53.7/46.2/0.1	54.8/45.2/0
PI	50/41.7/8.3	60/30/10	0/100/0	0/0/0	47.6/50/2.4
TE	44.4/50/5.6	40.7/59.3/0	0/100/0	0/0/0	30.9/63/6.2

S, Sensitive; R, Resistant; I, Intermediate.

### Trend visualization of resistance patterns over time

**FIGURE 4:**
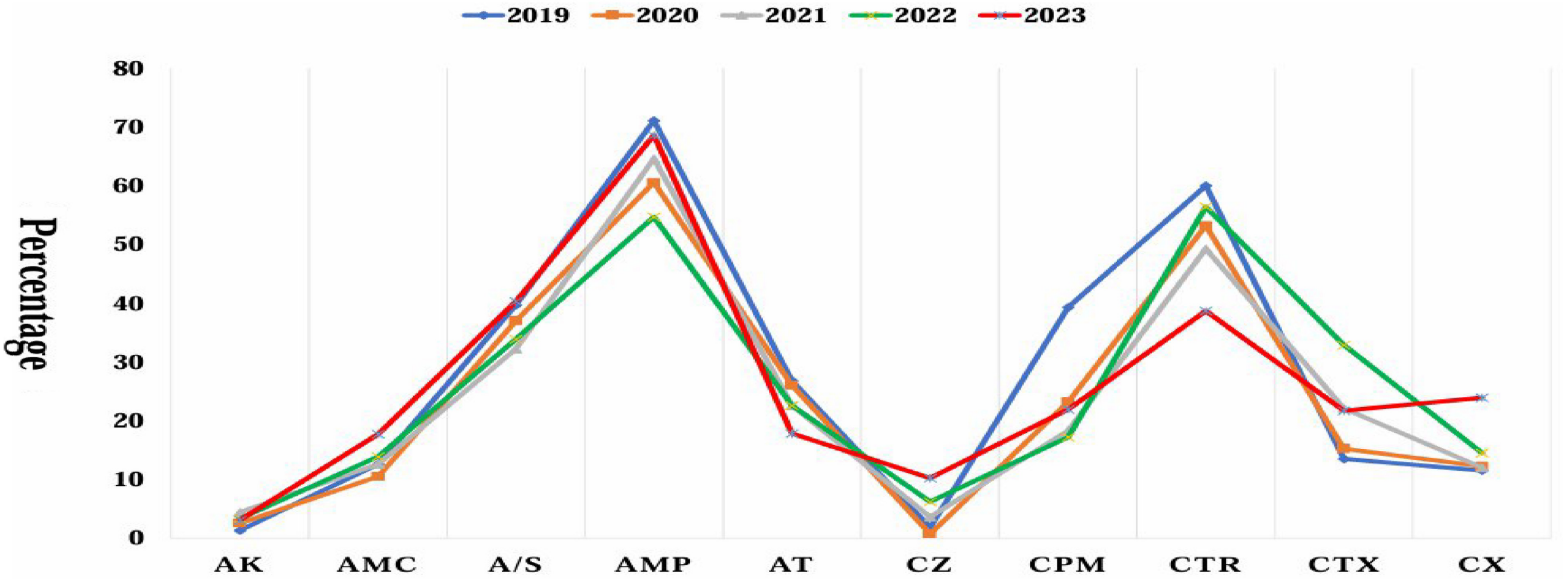
The resistance trend of *E. coli* against antibiotics most frequently used for treating UTI during the study period of 2019–2023.

**FIGURE 5:**
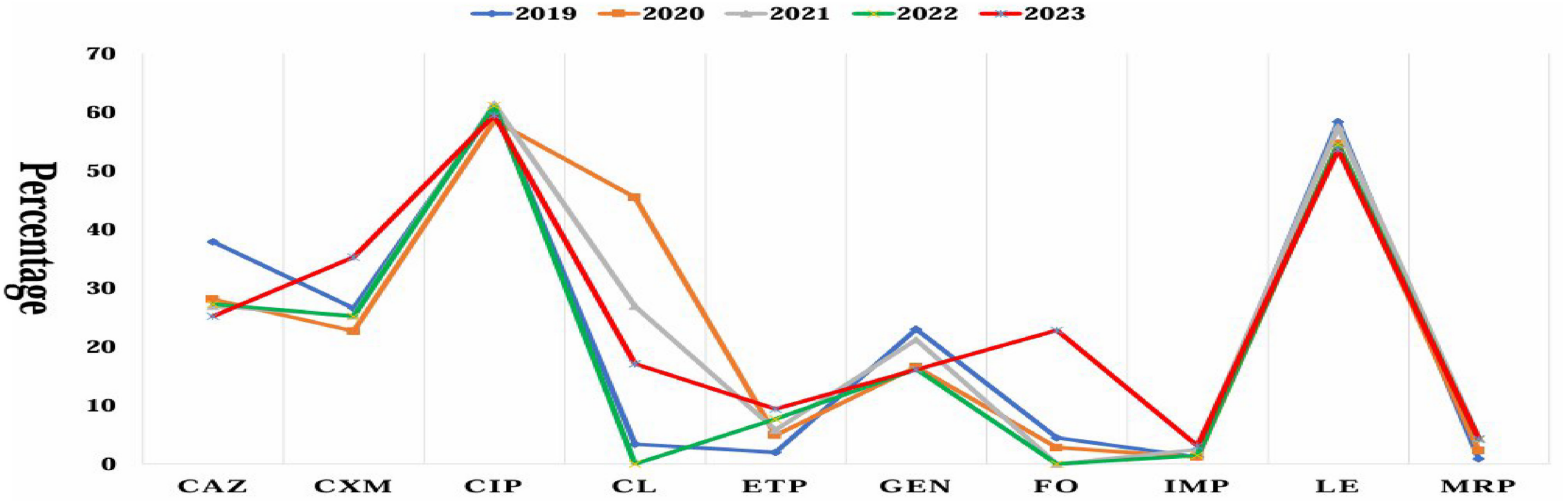
The resistance trend of *E. coli* against antibiotics most frequently used for treating UTI during the study period of 2019–2023.

**FIGURE 6:**
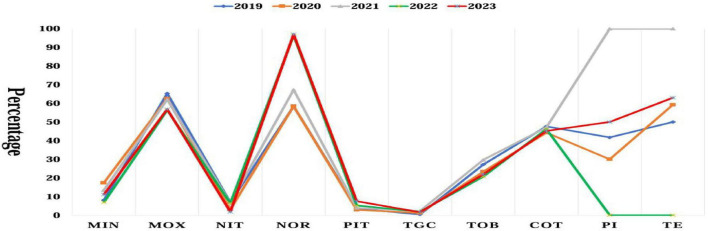
The resistance trend of *E. coli* against antibiotics most frequently used for treating UTI during the study period of 2019–2023.

Multidrug-resistant *E. coli* developed unusual patterns of resistance over time because it increased from 3.4% in 2019 to 45.5% in 2020 before becoming undetectable in 2022 and reclaiming 17.1% detection in 2023 (Figures 4, 5). The resistance patterns of polymyxin B and tetracycline followed tremendous fluctuations as polymyxin B reached 100% resistance in 2021 but returned to zero resistance in 2022 before climbing to 50% in 2023. The frequency of tetracycline resistance increased to reach its peak of 100% in 2021 before becoming completely absent in 2022 until it reemerged in 2023 at a level of 63%. These variations warrant further investigation (Figure 6). Resistance is increasing for all three antibiotic classes, including carbapenems, cephalosporins, and fluoroquinolones. The increasing resistance detected for the antimicrobial agents norfloxacin, cefotaxime, and ertapenem constitutes a major healthcare problem. The resistance levels of nitrofurantoin and ceftazidime antibiotics decreased, but polymyxins and colistin antibiotics demonstrated sporadic resistance patterns. It is essential to maintain both ongoing monitoring efforts and antimicrobial control practices to stop resistance development from getting more advanced (Table 2).

### Distribution of MDR and XDR

**FIGURE 7:**
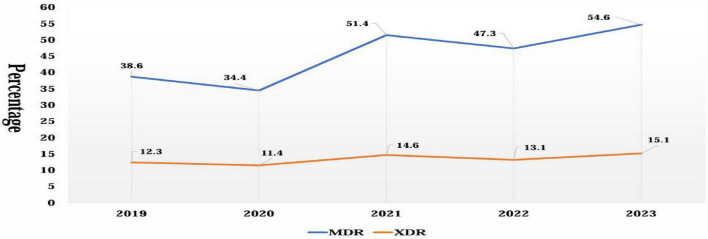
Distribution of MDR and XDR *E. coli* during the study period of 2019–2023.

The number of MDR isolates increased from 151 in 2019 to 232 in 2023. The total number of MDR isolates over the study period was 841. XDR isolates showed year-wise variation, with 86 in 2019, 59 in 2020, 114 in 2021, 99 in 2022, and 92 in 2023. The total number of XDR isolates was 450 (Figure 7).

Age-wise analysis demonstrated that MDR and XDR isolates were identified across all age groups during the study period, with comparatively higher proportions observed among elderly patients, particularly in the 61–80 and 81–100 years age groups. The highest MDR and XDR proportions were recorded in the 81–100 years group in 2019. An increasing trend in MDR isolates was observed in certain age groups over time, while the overall highest number of MDR isolates was documented in 2023 (Table 3).

**TABLE 3:** Distribution of MDR and XDR cases across different age groups (2019–2023).

Age in years	2019		2020		2021		2022		2023	
	MDR	XDR	MDR	XDR	MDR	XDR	MDR	XDR	MDR	XDR
0–20	5 (3.3)	6 (7.0)	7 (5.8)	3 (5.1)	11 (7.1)	11 (9.6)	18 (9.8)	9 (9.1)	23 (9.9)	11 (12.0)
21–40	23 (15.2)	17 (19.8)	13 (10.8)	9 (15.3)	26 (16.9)	21 (18.4)	27 (14.7)	14 (14.1)	38 (16.4)	17 (18.5)
41–60	31 (20.5)	16 (18.6)	37 (30.8)	17 (28.8)	31 (20.1)	27 (23.7)	51 (27.7)	23 (23.2)	52 (22.4)	24 (26.1)
61–80	35 (23.2)	27 (31.4)	32 (26.7)	14 (23.7)	37 (24.0)	33 (28.9)	46 (25.0)	31 (31.3)	47 (20.3)	21 (22.8)
81–100	48 (31.8)	17 (19.8)	29 (24.2)	16 (27.1)	42 (27.3)	19 (16.7)	37 (20.1)	20 (20.2)	45 (19.4)	13 (14.1)
> 100	9 (6.0)	3 (3.5)	2 (1.7)	0 (0.0)	7 (4.6)	3 (2.6)	5 (2.7)	2 (2.0)	27 (11.6)	6 (6.5)
Total	151 (100)	86 (100)	120 (100)	59 (100)	154 (100)	114 (100)	184 (100)	99 (100)	232 (100)	92 (100)

MDR, Multidrug resistant; XDR, Extensively drug-resistant.

Similarly, department-wise analysis indicated that MDR and XDR isolates were predominantly associated with inpatient department (IPD) samples throughout the study period. OPD samples also contributed substantially to resistant isolates, whereas EXT and EMR departments showed comparatively lower proportions of MDR and XDR isolates (Table 4).

**TABLE 4:** Distribution of MDR and XDR cases across different patient categories (2019–2023).

Category	2019		2020		2021		2022		2023	
	MDR	XDR	MDR	XDR	MDR	XDR	MDR	XDR	MDR	XDR
IPD	89 (58.9)	47 (54.7)	63 (52.5)	27 (45.8)	87 (56.5)	47 (41.2)	114 (62.0)	53 (53.5)	131 (56.5)	57 (62.0)
OPD	57 (37.7)	37 (43.0)	51 (42.5)	32 (54.2)	61 (39.6)	65 (57.0)	66 (35.9)	45 (45.5)	97 (41.8)	35 (38.0)
EXT	1 (0.7)	0 (0.0)	2 (1.7)	0 (0.0)	4 (2.6)	2 (1.8)	4 (2.2)	1 (1.0)	4 (1.7)	0 (0.0)
EMR	4 (2.6)	2 (2.3)	4 (3.3)	0 (0.0)	2 (1.3)	0 (0.0)	0 (0.0)	0 (0.0)	0 (0.0)	0 (0.0)
Total	151 (100%)	86 (100%)	120 (100%)	59 (100%)	154 (100%)	114 (100%)	184 (100%)	99 (100%)	232 (100%)	92 (100%)

IPD, Inpatient Department; OPD, Outpatient Department; EXT, External; EMR, Emergency Department.

**FIGURE 8:**
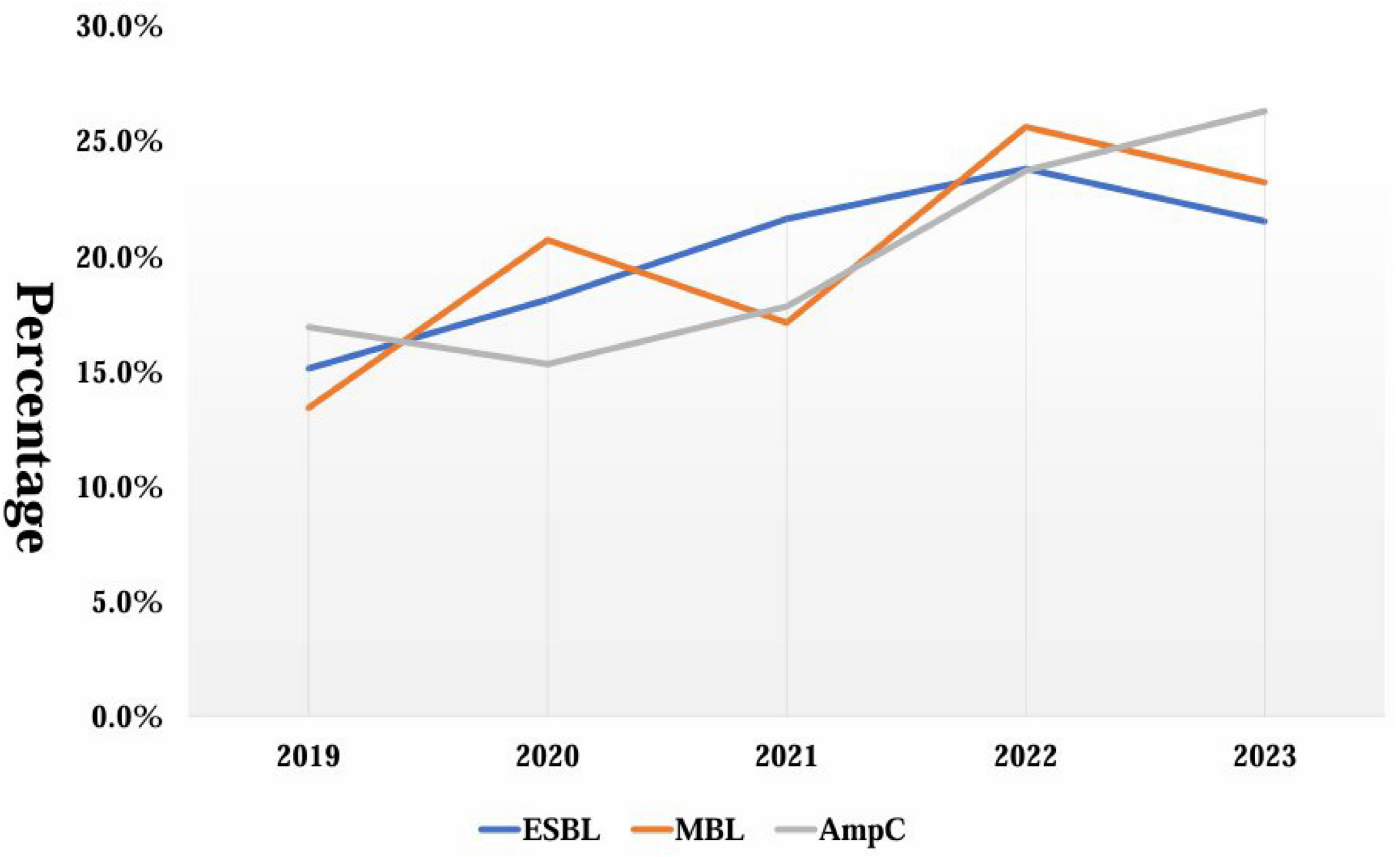
Trends in ESBL, MBL, and AmpC β-lactamase production among *E. coli* (2019–2023).

The number of ESBL-producing isolates increased from 106 cases in 2019 to 167 cases in 2022, followed by a slight decrease to 151 cases in 2023. MBL-producing isolates showed variation during the study period, with the highest number observed in 2022 (21 isolates) and 19 isolates recorded in 2023. AmpC-producing isolates increased from 86 cases in 2019 to 134 cases in 2023, with higher frequencies observed during the later study years (Figure 8).

ESBL-producing isolates showed variation across age groups, with a notable increase in the 41–60 years group from 2019 to 2022, followed by a slight decline in 2023. Overall, higher proportions were observed in the 61–80 years age group during the study period. MBL-producing isolates were also more frequently detected in the 61–80 years category, while AmpC producers showed an increasing trend over time, particularly in the 41–60 and 61–80 years groups (Table 5).

**TABLE 5:** Distribution of ESBL, MBL, and AmpC cases across different age groups (2019–2023).

Age in years	2019			2020			2021			2022			2023		
	ESBL	MBL	AmpC	ESBL	MBL	AmpC	ESBL	MBL	AmpC	ESBL	MBL	AmpC	ESBL	MBL	AmpC
0–20	6 (5.7)	0 (0.0)	9 (10.5)	8 (6.3)	0 (0.0)	11 (14.1)	17 (11.2)	0 (0.0)	17 (18.7)	15 (9.0)	0 (0.0)	13 (10.7)	23 (15.2)	1 (5.3)	14 (10.4)
21–40	21 (19.8)	3 (27.3)	21 (24.4)	21 (16.5)	2 (11.8)	11 (14.1)	43 (28.3)	3 (21.4)	21 (23.1)	31 (18.6)	4 (19.0)	27 (22.3)	27 (17.9)	5 (26.3)	26 (19.4)
41–60	20 (18.9)	1 (9.1)	32 (37.2)	39 (30.7)	3 (17.6)	19 (24.4)	34 (22.4)	6 (42.9)	32 (35.2)	57 (34.1)	7 (33.3)	35 (28.9)	48 (31.8)	5 (26.3)	37 (27.6)
61–80	33 (31.1)	4 (36.4)	17 (19.8)	33 (26.0)	10 (58.8)	27 (34.6)	37 (24.3)	2 (14.3)	11 (12.1)	46 (27.5)	9 (42.9)	29 (24.0)	33 (21.9)	4 (21.1)	42 (31.3)
81–100	24 (22.6)	2 (18.2)	7 (8.1)	24 (18.9)	2 (11.8)	9 (11.5)	19 (12.5)	2 (14.3)	8 (8.8)	16 (9.6)	1 (4.8)	13 (10.7)	19 (12.6)	4 (21.1)	11 (8.2)
> 100	2 (1.9)	1 (9.1)	0 (0.0)	2 (1.6)	0 (0.0)	1 (1.3)	2 (1.3)	1 (7.1)	2 (2.2)	2 (1.2)	0 (0.0)	4 (3.3)	1 (0.7)	0 (0.0)	4 (3.0)
Total	106 (100)	11 (100)	86 (100)	127 (100)	17 (100)	78 (100)	152 (100)	14 (100)	91 (100)	167 (100)	21 (100)	121 (100)	151 (100)	19 (100)	134 (100)

ESBL, Extended spectrum beta lactamases; MBL, Metallo beta lactamases; AmpC, Ampicillin class C beta lactamases.

Department-wise analysis indicated that ESBL-producing isolates were more commonly associated with IPD samples, with a peak observed in 2021, while OPD samples showed an increasing contribution in 2023. MBL-producing isolates were also predominantly found in IPD and OPD settings, whereas AmpC-producing isolates demonstrated a gradual increase in both departments over the study period. EXT samples showed comparatively lower frequencies of resistant isolates, and EMR samples demonstrated minimal detection in later years (Table 6).

**TABLE 6:** Distribution of ESBL, MBL and AmpC cases across different patient categories (2019–2023).

Category	2019			2020			2021			2022			2023		
	ESBL	MBL	AmpC	ESBL	MBL	AmpC	ESBL	MBL	AmpC	ESBL	MBL	AmpC	ESBL	MBL	AmpC
IPD	53 (50.0)	8 (72.7)	47 (54.7)	69 (54.3)	8 (47.1)	30 (38.5)	87 (57.2)	4 (28.6)	58 (63.7)	83 (49.7)	9 (42.9)	64 (52.9)	62 (41.1)	4 (21.1)	73 (54.5)
OPD	49 (46.2)	3 (27.3)	37 (43.0)	47 (37.0)	8 (47.1)	37 (47.4)	56 (36.8)	8 (57.1)	29 (31.9)	80 (47.9)	11 (52.4)	53 (43.8)	84 (55.6)	12 (63.2)	59 (44.0)
EXT	1 (0.9)	0 (0.0)	0 (0.0)	4 (3.1)	1 (5.9)	4 (5.1)	6 (3.9)	2 (14.3)	3 (3.3)	4 (2.4)	1 (4.8)	4 (3.3)	5 (3.3)	3 (15.8)	2 (1.5)
EMR	3 (2.8)	0 (0.0)	2 (2.3)	7 (5.5)	0 (0.0)	7 (9.0)	3 (2.0)	0 (0.0)	1 (1.1)	0 (0.0)	0 (0.0)	0 (0.0)	0 (0.0)	0 (0.0)	0 (0.0)
Total	106 (100)	11 (100)	86 (100)	127 (100)	17 (100)	78 (100)	152 (100)	14 (100)	91 (100)	167 (100)	21 (100)	121 (100)	151 (100)	19 (100)	134 (100)

P.C, Patient categories; ESBL, Extended spectrum beta lactamases; MBL, Metallo beta lactamases; AmpC, Ampicillin class C beta lactamases.

Out of 703 *Escherichia coli* isolates identified as ESBL producers and 82 as MBL producers, the most prevalent ESBL gene was *blaCTX-M*, found in 55.5% of isolates. This was followed by *blaTEM* (27.3%) and *blaSHV* (10.8%). Co-expression of ESBL genes was frequently observed, with *blaCTX-M + blaTEM* detected in 22.8% and *blaCTX-M + blaSHV* in 17.1% of isolates.

Among the 82 MBL-producing isolates, the most common gene was *blaNDM* (28.4%), followed by *blaOXA-48* (24.3%) and *blaVIM* (8.5%). Dual combinations were notably present, including *blaNDM + blaOXA-48* (8.5%) and *blaVIM + blaOXA-48* (7.3%). Additionally, triplet combinations indicating extensive drug resistance were detected, such as *blaVIM + blaNDM + blaOXA-48* (3.7%) and *blaKPC + blaNDM + blaOXA-48* (2.4%). These findings highlight a significant burden of multidrug resistance, with widespread circulation of high-risk β-lactamase genes in clinical *E. coli* isolates (Table 7).

**TABLE 7:** Prevalence of β-lactamase genes in *Escherichia coli* isolates from the Aseer region.

Gene/combination	ESBL (*n* = 703)	%	MBL (*n* = 82)	%
*blaCTX-M*	390	55.5	–	–
*blaSHV*	76	10.8	–	–
*blaTEM*	192	27.3	–	–
*blaKPC*	–	–	3	3.7
*blaVIM*	–	–	7	8.5
*blaNDM*	–	–	23	28.4
*blaOXA-48*	–	–	20	24.3
*blaCTX-M + blaSHV*	120	17.1	–	–
*blaCTX-M + blaTEM*	160	22.8	–	–
*blaSHV + blaTEM*	50	7.1	–	–
*blaKPC + blaVIM*	–	–	2	2.4
*blaKPC + blaNDM*	–	–	4	4.9
*blaKPC + blaOXA-48*	–	–	2	2.4
*blaVIM + blaNDM*	–	–	5	6.1
*blaVIM + blaOXA-48*	–	–	6	7.3
*blaNDM + blaOXA-48*	–	–	7	8.5
*blaKPC + blaNDM + blaOXA-48*	–	–	2	2.4
*blaVIM + blaNDM + blaOXA-48*	–	–	3	3.7
*blaKPC + blaVIM + blaOXA-48*	–	–	1	1.2
*blaKPC + blaVIM + blaNDM*	–	–	1	1.2

## Discussion

According to the present study, female patients consistently exhibited a higher occurrence of UTIs than male patients throughout 2019–2023. This finding is in agreement with previous epidemiological studies reporting that females are more susceptible to UTIs because of anatomical, physiological, and behavioral factors. The shorter female urethra and its proximity to the anal region facilitate the ascent of uropathogens such as *E. coli* into the urinary tract. In addition, pregnancy-associated urinary stasis and vesicoureteral reflux, as well as postmenopausal estrogen deficiency leading to altered vaginal flora, may further increase susceptibility to recurrent infections. Behavioral factors such as sexual activity, contraceptive use, and inadequate hygiene practices may also contribute to the increased prevalence observed among females. Similar female predominance has been reported in studies from Saudi Arabia and India, where females accounted for 62.6, 64.04, and 60.7% of UTI cases, respectively (Alhazmi et al., 2023; Alrashid et al., 2022; Bhargava et al., 2022). The persistent predominance of female patients in the present study highlights the importance of targeted preventive measures, including awareness regarding hygiene, early symptom recognition, and appropriate clinical management in high-risk groups.

The present study also demonstrated fluctuations in IPD and OPD UTI cases during the study period. A decline in hospital-based cases during 2020–2021 was observed, which may be associated with restricted healthcare access and reduced hospital attendance during the COVID-19 pandemic. The subsequent rise in cases after 2021 possibly reflects the restoration of healthcare services and delayed patient consultations following lockdown periods (Birkmeyer et al., 2020; Moynihan et al., 2021). Similarly, OPD cases showed a marked decline during the pandemic years, followed by a substantial increase in 2023, likely due to normalization of healthcare-seeking behavior and improved accessibility to outpatient services (Mehrotra et al., 2020; World Health Organization [WHO], 2022). These findings indicate the considerable impact of the COVID-19 pandemic on healthcare utilization patterns in patients with UTIs.

Age-wise analysis in the current study revealed that individuals aged 61–80 years had the highest occurrence of UTIs during most years of the study period. Elderly individuals are known to be at greater risk of UTIs because of age-related immune decline, urinary retention, catheterization, and associated comorbidities (Rowe and Juthani-Mehta, 2013). An increased proportion of cases among patients aged 81–100 years during 2020 may be linked to the greater vulnerability of older adults during the COVID-19 pandemic and increased hospitalization rates in this population (Mueller et al., 2020). Furthermore, the rise in UTI cases among individuals aged 61–80 years during 2023 may reflect increasing comorbid conditions and possible antimicrobial resistance patterns in elderly patients (Moynihan et al., 2021). In contrast, younger age groups showed comparatively lower UTI prevalence throughout the study period (Shaikh et al., 2008).

Overall, the findings of the present study emphasize that female gender and elderly age groups remain major risk factors associated with UTIs in the studied population. The observed temporal variations in OPD and IPD cases further demonstrate the influence of healthcare accessibility and pandemic-related disruptions on disease reporting and management. These findings support the need for focused preventive strategies, early diagnosis, rational antibiotic use, and improved healthcare accessibility, particularly for elderly and high-risk female patients.

The present study demonstrated a progressive increase in antimicrobial resistance among *E. coli* isolates from 2019 to 2023, highlighting the growing therapeutic challenges associated with urinary tract infections. Carbapenem resistance, although relatively low compared with other antibiotic groups, showed a gradual increase during the study period, with imipenem resistance rising from 1.2 to 3.1%, meropenem from 0.9 to 4.3%, and ertapenem from 2 to 9.4%. These findings suggest the possible emergence of carbapenem-resistant *E. coli* strains in the study population. Previous studies have associated carbapenem resistance with carbapenemase-producing genes such as *blaKPC*, *blaNDM*, *blaOXA-48*, and *blaVIM* (Venne et al., 2023).

Among aminoglycosides, amikacin retained comparatively high effectiveness throughout the study period, with resistance rates ranging from 1.3 to 3.1%, whereas gentamicin resistance varied between 16 and 23%. Tobramycin resistance peaked in 2021 and subsequently declined by 2023, possibly reflecting changes in antibiotic usage practices and antimicrobial stewardship measures (Devnikar et al., 2024). The relatively lower resistance observed for aminoglycosides in the present study suggests their continued usefulness in the management of MDR *E. coli* infections. Although the primary focus of this study was β-lactamase-mediated resistance, aminoglycoside resistance mechanisms were discussed to provide additional insight into the broader multidrug resistance profile of the isolates, as co-resistance to aminoglycosides is frequently associated with β-lactam-resistant strains. Previous studies have linked aminoglycoside resistance to aminoglycoside-modifying enzymes such as *aac(6’)-Ib* and *aph(3’)-VI*; however, these determinants were not specifically investigated in the present study.

Cephalosporin resistance showed notable fluctuations during the study period. Cefotaxime resistance increased to 32.9% in 2022, then declined in 2023, while ceftriaxone resistance reached 56.4% in 2022 and later decreased to 38.7%. These findings may reflect variations in antibiotic prescribing practices and infection control interventions over time. The high resistance rates observed in the present study are consistent with the widespread occurrence of ESBL-producing *E. coli* strains reported globally. Previous literature suggests that genes such as *blaCTX-M*, *blaSHV*, and *blaTEM* may contribute to this resistance pattern.

Fluoroquinolone resistance remained consistently high throughout the study period, with ciprofloxacin and levofloxacin resistance exceeding 50% in most years. A marked increase in norfloxacin resistance was also observed, reaching 97.1% in 2023. These findings indicate a substantial reduction in the clinical utility of fluoroquinolones for empirical treatment of uncomplicated UTIs in the studied population. Previous reports have associated fluoroquinolone resistance with plasmid-mediated quinolone resistance determinants and mutations in *gyrA* and *parC* genes (Kim et al., 2009). However, since molecular analysis was not performed in the present study, these mechanisms can only be considered possible explanations for the observed resistance trends.

Resistance to β-lactam/β-lactamase inhibitor combinations also increased during the study period. Amoxicillin-clavulanate resistance increased from 12.6 to 17.7%, while piperacillin-tazobactam resistance rose from 3.5 to 7.5%. The increasing resistance observed in the current study may be associated with enhanced β-lactamase production and other adaptive resistance mechanisms described in previous studies (Zhang et al., 2025). However, these mechanisms were not specifically investigated in the present work.

Tetracycline resistance showed variability during the study period, with a temporary rise in 2020 followed by a decline in 2023. In contrast, tigecycline maintained very low resistance rates, indicating preserved activity against MDR *E. coli* isolates. Earlier studies have linked tetracycline resistance to efflux pump genes such as *tetA* and *tetB* (Karami et al., 2006), although these genetic determinants were not analyzed in this study.

In this study, the resistance rate of nitrofurantoin decreased notably from 6.8 to 1.8%, reinforcing its continued role as the primary oral therapeutic option for uncomplicated UTIs. Similarly, polymyxin resistance patterns showed atypical fluctuations, with colistin resistance peaking at 45.5% in 2020 and polymyxin B demonstrating a transient 100% resistance in 2021 before declining thereafter. These variations may, in part, reflect the influence of the antimicrobial stewardship program that was active during the study period, alongside infection control measures and the sporadic emergence of resistant strains (Venne et al., 2023). Continued surveillance is therefore essential to better understand such trends. Together with strengthened antimicrobial stewardship practices and consideration of alternative therapeutic options, these measures are urgently required to control multidrug-resistant *E. coli* in urinary tract infections. The global rise in resistance to carbapenems, fluoroquinolones, and polymyxins further underscores the need for coordinated local and international public health responses. The present study demonstrated a concerning increase in MDR and XDR *E. coli* isolates associated with UTIs between 2019 and 2023. MDR cases increased from 151 in 2019 to 232 in 2023, with a marked rise observed after 2021, indicating a progressive decline in the effectiveness of commonly used antimicrobial agents. Although XDR isolates showed some year-to-year variation, their numbers remained consistently high throughout the study period. The overall detection of 841 MDR and 450 XDR isolates during 5 years highlights the substantial burden of antimicrobial resistance in the studied population and emphasizes the need for strengthened antimicrobial stewardship and infection control measures.

The increase in resistant isolates observed in the present study may also reflect the indirect impact of the COVID-19 pandemic on antimicrobial usage and healthcare practices. During the pandemic, empirical and prolonged antibiotic administration was frequently practiced to prevent or manage suspected secondary bacterial infections, which may have contributed to increased selective pressure for resistant organisms. Similar observations have been reported in studies from the Middle East, including Iran, where increased antibiotic consumption and healthcare-related challenges during the pandemic were associated with rising antimicrobial resistance trends (Khoshbakht et al., 2022).

Age-wise analysis in the current study revealed that MDR and XDR *E. coli* infections were more common among elderly patients, particularly those aged 61–100 years. Older individuals are more likely to have repeated healthcare exposure, prolonged hospitalization, catheterization, and multiple comorbidities, all of which may contribute to the emergence and persistence of resistant infections (Madrazo et al., 2021). A gradual increase in MDR isolates was also observed in younger age groups during 2023, suggesting a possible expansion of resistant strains beyond traditionally high-risk populations.

Hospitalized patients accounted for the majority of MDR and XDR cases in the present study, with IPD isolates showing the highest resistance burden during 2022–2023. This finding supports the association between hospital settings and increased antimicrobial resistance, likely due to higher antibiotic exposure and greater circulation of resistant organisms within healthcare environments. In comparison, OPD cases fluctuated during the study period, while EXT and EMR cases remained relatively low.

The study also demonstrated a progressive increase in β-lactamase-producing *E. coli* isolates. ESBL-producing isolates increased steadily and reached their highest number in 2022, while AmpC-producing isolates also showed a gradual rise from 2019 to 2023 (Raphael et al., 2021). These findings indicate the growing prevalence of resistant phenotypes among uropathogenic *E. coli* in the studied region. The higher occurrence of ESBL-producing isolates among elderly patients further supports the association between advanced age, repeated antibiotic exposure, and resistant infections (Alkan et al., 2024).

Among the MBL-producing bacterial isolates, the population levels were low, but the tributary data from 2020 and 2022 showed maximum incidence in the 61–80 age cohort, respectively, with estimated proportions of 58.8 and 42.9%. A meta-analysis from the Gulf Cooperation Council has detected MBL-producing *E. coli* strains mostly with *CTX-M* enzymatic properties, which create substantial treatment dilemmas (Bindayna et al., 2022). Beta-lactamase-producing AmpC isolates displayed an increasing trend between 2017 and 2023, specifically among individuals between 41 and 60 years old and those between 61 and 80 years who reached their highest point with 27.6% in the 41–60 age group and 31.3% in the 61–80 age group in 2023. Middle-aged and elderly populations require specific infection control surveillance because these resistant microbial strains continue to increase in numbers.

ESBL-producing isolates predominantly affected hospitalized patients (IPD) during 2021 when they reached their highest levels at 57.2% before declining to 41.1% in 2023. ESBL cases diagnosed from patients visiting OPD clinics showed continuous growth, resulting in 55.6% of all recorded cases during 2023. The COVID-19 pandemic likely played a role in driving the change of ESBL-producing *E. coli* from inpatients toward outpatients from 2021 until 2023. The combination of wide-scale early antibiotic use and interrupted hospital infection control, besides resistance development during that period, likely triggered the early IPD increase. The reduction of IPD cases occurred through better hospital antibiotic stewardship, but OPD cases grew because of antibiotic overuse by the public, combined with delayed medical care and self-treatment. The detection rate increased through better outpatient diagnostic capabilities (Mai and Espinoza, 2023). The percentage of MBL producers initially reached its highest levels in IPD patients during 2019 (72.7%) before OPD patients displayed the highest proportion in 2023 (63.2%). The recent observation shows that highly resistant strains successfully spread into public communities, which demands comprehensive antimicrobial stewardship alongside strong public health measures.

Analysis of AmpC beta-lactamase production in IPD patients demonstrates stable prevalence, which reached the highest level in 2021 (63.7%) and exhibited slight fluctuations afterward. AmpC beta-lactamase production within OPD patients increased from 43.0% in 2019 until it reached 44.0% in 2023. Extended care (EXT) settings displayed low levels of resistant isolates, except for MBL producers, which increased to 15.8% during 2023. After the 2021 emergency (EMR) cases eliminated both MBL and AmpC-producing isolates were eliminated from their samples.

Middle Eastern populations experience a growing threat of beta-lactamase-producing *E. coli* infections in healthcare facilities and community settings. Thus, it is vital to implement better infection control practices to continuously monitor resistant pathogens and implement successful antimicrobial stewardship initiatives to stop their spread.

The emergence of ESBL and metallo-β-lactamase (MBL) producing *Escherichia coli* strains is a growing global concern, with the Middle East showing alarming trends that mirror the worldwide pattern. The predominance of the *blaCTX-M* gene, detected in 55.5% of ESBL-producing isolates in the present data, aligns with numerous regional and international studies. For instance, research from Saudi Arabia has demonstrated the dominance of the *CTX-M* variant in ESBL-producing *E. coli*, with studies revealing that up to 33.3% of such isolates carried the *blaCTX-M* gene (Ibrahim et al., 2021). Similar findings from Qatar reported that 87.8% of *E. coli* and *Klebsiella pneumoniae* isolates from pediatric populations harbored *blaCTX-M-15* (Perez-Lopez et al., 2020). These figures highlight the widespread nature of this gene and suggest strong selective pressure likely due to extensive cephalosporin use in clinical settings.

The co-expression of ESBL genes, such as *blaCTX-M* with *blaTEM* (22.8%) and *blaCTX-M* with *blaSHV* (17.1%), reflects a complex resistance mechanism that limits therapeutic choices. Co-harboring of multiple ESBL genes in *E. coli* has been well-documented in countries like Egypt, where genetic exchange through mobile plasmids contributes to the rapid dissemination of these resistance determinants (Hassuna et al., 2020).

Among the MBL-producing isolates, the most frequent gene was *blaNDM* (28.4%), followed closely by *blaOXA-48* (24.3%) and *blaVIM* (8.5%). These findings are in line with regional reports; for example, in Iran, high rates of *blaNDM-1* and *blaOXA-48* co-expression were found in carbapenem-resistant *Enterobacterales* (Mokhtari et al., 2022). In Lebanon, the presence of *blaOXA-48* increased markedly in clinical isolates over a few years, indicating local clonal expansion and inadequate containment practices (Hammoudi et al., 2014). The presence of dual and triple MBL gene combinations, including *blaNDM + blaOXA-48* (8.5%), *blaVIM + blaOXA-48* (7.3%), and even extensive drug resistance combinations such as *blaVIM + blaNDM + blaOXA-48* (3.7%), illustrates the dangerous trajectory toward pan-drug resistance. The detection of *blaKPC + blaNDM + blaOXA-48* (2.4%) is particularly alarming, as it reflects the convergence of class A, B, and D β-lactamases, potentially rendering all β-lactam antibiotics ineffective.

Globally, these gene combinations are becoming increasingly common due to the movement of resistance plasmids across borders via human travel, food chains, and environmental reservoirs. The World Health Organization (WHO) has identified ESBL- and carbapenemase-producing *E. coli* as critical priority pathogens for research and drug development (World Health Organization [WHO], 2017).

The spread of these genes can be attributed to several factors, including overuse and misuse of antibiotics, lack of robust antimicrobial stewardship programs, and inadequate infection control measures in hospitals. Studies from Middle Eastern countries have emphasized the urgent need for enhanced national surveillance systems to track resistance trends and the genetic epidemiology of high-risk clones (Alharazi et al., 2024; Al-Zarouni et al., 2008; Sonnevend et al., 2022). In addition, regional data underscore the critical role of community-acquired infections in perpetuating resistance, further complicating infection control.

Efforts to combat this growing threat must focus on integrated strategies. This includes strengthening laboratory capacity for molecular diagnostics, implementing strict antibiotic prescribing policies, and enhancing hospital hygiene practices. Public education campaigns are essential to reduce self-medication and improve awareness about antimicrobial resistance. Furthermore, continued research into the genetic mechanisms and transmission pathways of these resistance genes is vital for developing novel therapeutic interventions.

## Limitations of the study

This study has certain limitations that should be acknowledged. First, as a retrospective study, comprehensive clinical outcome and follow-up data were not available, which limited the ability to correlate antimicrobial resistance profiles with patient treatment outcomes. Second, molecular analysis was confined to the major clinically relevant and commonly prevalent ESBL and carbapenemase genes; other resistance determinants, including genes associated with fluoroquinolone and aminoglycoside resistance, were not investigated. Finally, the study was primarily descriptive in nature, and no inferential statistical analyses were performed. Therefore, potential associations between demographic variables and antimicrobial resistance patterns could not be statistically validated.

## Conclusion

This study demonstrates an increasing trend of urinary tract infections (UTIs) that affects primarily females and elderly patients, together with the predominance of Escherichia coli as the leading uropathogen. Research data show a continuous growth of antimicrobial resistance mechanisms, which primarily target fluoroquinolones, along with cephalosporins and β-lactam/β-lactamase inhibitor combination antibiotics. The distribution of multidrug-resistant *E. coli* strains along with extensively drug-resistant strains and β-lactamase-producing pathogens with ESBL, MBL and AmpC enzymes creates substantial difficulties regarding therapy and public health responses.

An alarming sign of community-wide carbapenemase gene distribution appears when experts detect *blaNDM, blaOXA-48*, and *blaKPC* genes in outpatient settings. The presence of multiple drug resistance genes creates severe limitations for available medical treatments, thereby requiring advanced molecular diagnostic systems and resistance-tracking programs.

Countermeasures should combine three pillars of strict antimicrobial control practices and routine resistance surveillance, along with improved infection containment systems. Effective public education about hygiene practices and rational antibiotic use is essential for controlling antibiotic resistance throughout the community. The research priority stands in genomic monitoring systems with quick detection methods alongside new therapeutic strategies for combating evolving UTI drug resistance threats.

## Data Availability

The original contributions presented in the study are included in the article/supplementary material, further inquiries can be directed to the corresponding author.
